# Molecular epidemiology of *Brucella* species in mixed livestock-human ecosystems in Kenya

**DOI:** 10.1038/s41598-021-88327-z

**Published:** 2021-04-23

**Authors:** James M. Akoko, Roger Pelle, AbdulHamid S. Lukambagire, Eunice M. Machuka, Daniel Nthiwa, Coletha Mathew, Eric M. Fèvre, Bernard Bett, Elizabeth A. J. Cook, Doreen Othero, Bassirou Bonfoh, Rudovick R. Kazwala, Gabriel Shirima, Esther Schelling, Jo E. B. Halliday, Collins Ouma

**Affiliations:** 1 Department of Biomedical Sciences and Technology, Maseno University, Kisumu, Kenya; 2 Biosciences Eastern and Central Africa-International Livestock Research Institute (BecA-ILRI) Hub KE, Nairobi, Kenya; 3 International Livestock Research Institute, Nairobi, Kenya; 4 Sokoine University of Agriculture, Morogoro, Tanzania; 5 Department of Biological Sciences, University of Embu, Embu, Kenya; 6 Institute of Infection, Veterinary and Ecological Sciences, University of Liverpool, Liverpool, UK; 7 Department of Public Health, Maseno University, Kisumu, Kenya; 8 Centre Suisse de Recherches Scientifiques en Côte d’Ivoire, Abidjan, Côte d’Ivoire; 9 Nelson Mandela African Institute of Science and Technology, Arusha, Tanzania; 10 Vétérinaires Sans Frontières Suisse, Bern, Switzerland; 11 Institute of Biodiversity, Animal Health and Comparative Medicine, College of Medical Veterinary and Life Sciences, University of Glasgow, Glasgow, G12 8QQ UK

**Keywords:** Policy and public health in microbiology, Bacterial infection

## Abstract

Brucellosis, caused by several species of the genus *Brucella*, is a zoonotic disease that affects humans and animal species worldwide. Information on the *Brucella* species circulating in different hosts in Kenya is largely unknown, thus limiting the adoption of targeted control strategies. This study was conducted in multi-host livestock populations in Kenya to detect the circulating *Brucella* species and assess evidence of host–pathogen associations. Serum samples were collected from 228 cattle, 162 goats, 158 sheep, 49 camels, and 257 humans from Narok and Marsabit counties in Kenya. Information on age, location and history of abortion or retained placenta were obtained for sampled livestock. Data on age, gender and location of residence were also collected for human participants. All samples were tested using genus level real-time PCR assays with primers specific for IS*711* and bcsp31 targets for the detection of *Brucella*. All genus positive samples (positive for both targets) were further tested with a speciation assay for *AlkB* and *BMEI1*162 targets, specific for *B. abortus* and *B. melitensis,* respectively. Samples with adequate quantities aggregating to 577 were also tested with the Rose Bengal Test (RBT). A total of 199 (33.3%) livestock and 99 (38.5%) human samples tested positive for genus *Brucella*. Animal *Brucella* PCR positive status was positively predicted by RBT positive results (OR = 8.3, 95% CI 4.0–17.1). Humans aged 21–40 years had higher odds (OR = 2.8, 95% CI 1.2–6.6) of being *Brucella* PCR positive compared to the other age categories. The data on detection of different *Brucella* species indicates that *B. abortus* was detected more often in cattle (OR = 2.3, 95% CI 1.1–4.6) and camels (OR = 2.9, 95% CI 1.3–6.3)*,* while *B. melitensis* was detected more in sheep (OR = 3.6, 95% CI 2.0–6.7) and goats (OR = 1.7, 95% CI 1.0–3.1). Both *B. abortus* and *B. melitensis* DNA were detected in humans and in multiple livestock host species, suggesting cross-transmission of these species among the different hosts. The detection of these two zoonotic *Brucella* species in humans further underpins the importance of One Health prevention strategies that target multiple host species, especially in the multi-host livestock populations.

## Introduction

The genus *Brucella* consists of several species of gram negative, facultative bacteria, causing brucellosis in humans, livestock, and wildlife hosts worldwide^1^. *Brucella abortus, B. melitensis* and *B. suis* are the most reported zoonotic species of *Brucella*^2^. Brucellosis causes reproductive disorders such as abortion, infertility, reduced milk yield and retained placenta in livestock, while humans suffer from a long and debilitating illness that is characterized by undulating fever^3^. Brucellosis in humans is largely due to transmission from infected animals through consumption of raw contaminated livestock products, particularly milk or through contact with secretions from infected animals, especially during parturition^4^. *Brucella* bacteria are highly infectious, thus posing an occupational hazard to persons involved in handling products from infected animals, including laboratory personnel, veterinarians, slaughter personnel and farmers who assist animals when giving birth^1^.

Brucellosis remains endemic or is a re-emerging neglected zoonosis in many parts of the world, especially Africa and Asia^5^. Brucellosis is emerging as a growing problem in intensive, peri-urban, small holder dairy production systems^6, 7^. Brucellosis has been successfully controlled in several developed countries using host species-specific livestock vaccines or test and slaughter policies in animals with no history of vaccination^8^. In Kenya, seroprevalences ranging from 0.1 to 46.5% in humans^9–11^ and 1.0–38.0% in livestock have been reported, with higher prevalence recorded in pastoral areas^9, 12, 13^.

While several serological studies conducted in animals and humans in many parts of sub-Saharan Africa revealed that *Brucella* antibodies are widespread, serology tests are unable to indicate which *Brucella* species were responsible for inducing antibodies in the host^14, 15^. A modelling framework that integrated serological data sets from northern Tanzania to determine the source of human infection, hypothesized that sheep and goats are the main source of human infection^16^. In Kenya, a strong association between human and animal *Brucella* seropositivity has also been reported^9^. However, information on the *Brucella* species circulating in the different hosts remains limited. This knowledge is key to understanding the epidemiology of brucellosis and could form the basis for the development of targeted control programs. Therefore, this study was conducted to identify the species of *Brucella* circulating in livestock and humans in the mixed livestock-human ecosystems in Narok and Marsabit in Kenya.

## Methods

### Study area

**Figure 1:**
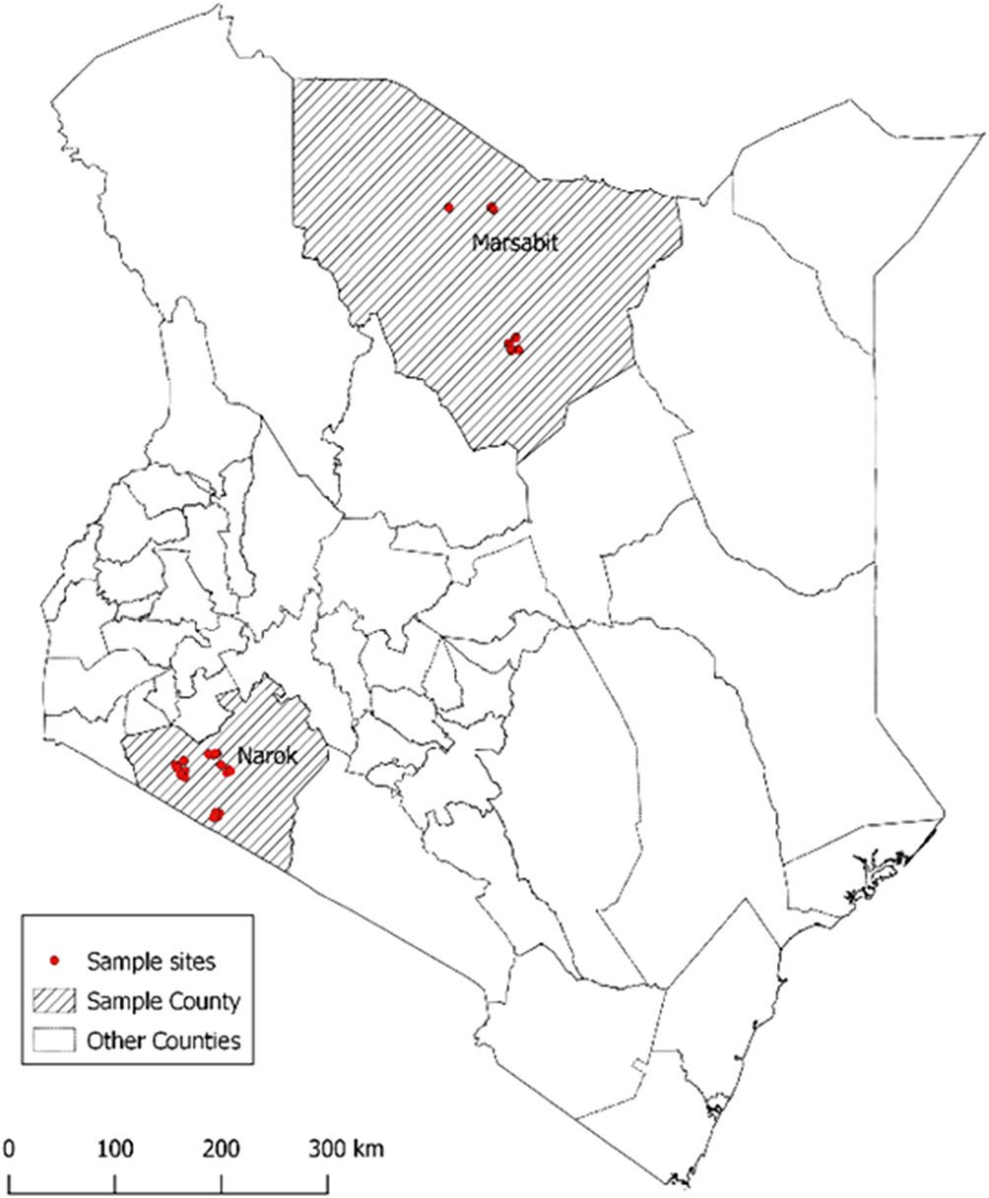
Map of Kenya showing the location of the study areas in Narok and Marsabit. The map was drawn by Fred Otieno using QGIS software, version 3.4.14–1 (http://www.gisagmaps.com/qgis-download/)^19^.

The study was conducted in pastoral areas of Narok and Marsabit counties in Kenya (Fig. 1). The two sites were purposefully selected due to high numbers of livestock kept in close contact with humans under a nomadic pastoralism system. Marsabit County has the highest reported *Brucella* spp. seroprevalence in humans and camels in Kenya^8, 9, 13^, while several serological studies have also demonstrated the exposure to *Brucella* spp. in Narok County^12, 13, 17^.

Narok County is located on the southern border of Kenya and Tanzania with an altitude of 1700–3000 m above sea level. It is a predominantly pastoral area with cattle, sheep and goats being kept in proximity with wildlife; Marsabit is the largest, most arid, and sparsely populated County in Kenya. Marsabit mostly comprises a vast lowland scrub desert ranging from 400 to 700 m above the sea level. Nomadic pastoralism that involves mixed herds of camels, cattle, sheep and goats is the main economic activity in Marsabit, practiced by up to 80% of the occupants^18^.

### Sample size determination

The sample size was calculated using the formula for detecting presence or absence of a disease^20^. Prevalence estimates from a previous study were assumed as 15.3% for humans, 3.3% for cattle, 3.6% for small ruminants (sheep and goats) in Narok and 46.5% for humans, 11.2% cattle, 16.1% small ruminants and 11.1% for camels in Marsabit^9^. The sample size calculated for each host was then adjusted to cater for potential clustering of *Brucella* species at the herd level using inter-cluster correlation coefficient (*icc*) of 0.4 and a sample size of 2.5 (1–5 animals) collected from each animal species at the herd level, which then translated into a design effect of 1.6. Therefore, a minimum of 228, 160, 40, 183 samples, from humans, small ruminants (sheep and goats), camels, and cattle, was estimated to be the total samples needed from the two sites.

### Study design and sampling strategy for animals

Data were collected through a cross-sectional study that targeted herds with current or previous clinical signs suggestive of brucellosis. The targeted study design was adopted to increase chances of getting animals with circulating *Brucella* DNA for subsequent species identification.

Animal health providers and public health officers working in Narok and Marsabit were consulted to identify four locations from each of the two sites with reported cases of illness in animals and or people with clinical signs suggestive of brucellosis. In each location, animal herds with suspected brucellosis or reported history of brucellosis within the past two years were identified and visited. A maximum of five adult animals were randomly selected for sampling from each species within the herd when present (cattle, sheep, goats, and camels). Blood was collected via jugular venipuncture into a plain 10 ml vacutainer tube. The samples were then labeled and transported in a cool box and ice packs (~ 4 °C) to the field laboratory, where they were centrifuged on the same day of collection at 5000 rpm for five minutes to obtain serum. The serum samples were transported at 4 °C and stored at − 20 °C until testing at the Biosciences East and Central Africa (BecA) laboratories, hosted at the International Livestock Research Institute (ILRI), Nairobi, Kenya.

### Sampling strategy for humans

The health care facilities visited by people living in and around the sampled livestock populations were identified. Three dispensaries in Narok (Oloolaimutia, Talek and Aitong) were visited and included in the study. The Marsabit County hospital, serving the entire county and one dispensary in North Horr Sub-County were included in the Marsabit study site. Patients referred to the laboratory at each facility for brucellosis testing, based on clinical suspicion, were approached, and consented for inclusion in this study. Venous blood was drawn from participants into a 6 ml plain vacutainer tube by a laboratory technologist working in the study health facilities, upon receiving written informed consent. The blood samples were kept upright at room temperature for not less than 10 min to facilitate clotting, then centrifuged at 5000 rpm for five minutes to obtain serum for routine serological testing of brucellosis at the hospital. A volume of approximately 1 ml extracted serum from each human subject was aliquoted into cryotubes for this study. All human sera were also transported at 4 °C and stored at − 20 °C until further molecular and serological testing at the BecA laboratory facility at ILRI, Nairobi, Kenya.

### Questionnaire administration

A brief structured questionnaire was administered to the head of each household visited for animal sampling to capture information on sex, location, history of abortion and retained placenta in livestock. For human sampling, information on the age, gender, and location of residence of each sampled human subject was recorded.

### Sample selection and testing rationale

This study is focused on the molecular epidemiology of *Brucella*. Therefore, the lab procedure begun with DNA extraction from all the samples for PCR testing to detect the genus *Brucella* and to identify *Brucella* spp. Samples with sufficient quantities were later selected for RBT testing to assess if RBT positive status could be used as a predictor for PCR positivity.

### DNA extraction and purification

Genomic DNA was extracted from all serum samples using QIAamp DNA mini kit, (QIAGEN Germany) as per manufacturer’s guidelines. Briefly, 20 µl of proteinase K was added to 200 µl serum, mixed with 200 µl of lysis buffer and left to digest at room temperature for 2 h. The lysate was then loaded into the spin columns (provided with the kit), and manufacturers guidelines followed, before eluting the genomic DNA in 50 µl of elution buffer. The DNA quality and quantity for each of the extracts derived from the original sera were assessed using a NanoDrop 2000c Spectrophotometer (ThermoFisher Scientific, USA) before being stored at − 20 °C until PCR was done.

### Real-time PCR

The PCR assays were first optimized to our local environmental conditions with reference to published standards (supplementary material S1). Each DNA extract was tested using primers targeting the *Brucella*-specific insertion sequence IS*711* to detect the genus *Brucella* as previously published^21^. A second assay was also run independently on all the samples with the bcsp31target, as adopted from a previous study to detect *Brucella* genus^22^. See supplementary table S2 for primer and probe details. Both quantitative PCR assays were performed using an ABI 7500 thermocycler machine (Applied Biosystems, Life Technologies, Singapore). All reaction mixtures (20 µl) were prepared by mixing 4 µl of DNA template with 0.25 µM of fluorescent probe, 0.5 µM of each of the primers and 10 µl of the Luna Universal Probe qPCR master mix (404 with UDG; New England BioLabs, MA, USA). All test runs were performed with the following PCR conditions; pretreatment with UDG 50 °C for 2 min, then polymerase activation and DNA denaturation at 95 °C for 10 min, followed by 40 cycles of 95 °C for 15 s of amplification step, then 1 min of annealing at 57 °C. The positive controls (DNA extracts from *Brucella* strains, *B. melitensis* 16M and *B. abortus* 544) used in this study were both sourced from the Friedrich-Loeffler-Institute *Brucella* Reference Laboratory in Germany). Positive controls, no template control and samples were all loaded in duplicates before sealing the plate in every run. A run was only considered valid if all negative/no-template controls did not amplify, and positive controls amplified within agreeable range of the standard curve equivalent. A test run was classified as valid when amplification was observed for positive controls and no amplification was observed for the negative controls.

All samples that showed amplification and a cycle threshold (Ct) value < 40 in one or both duplicate wells were considered assay positive. Samples positive in both the IS*711* and bcsp31 assays were classified as *Brucella* genus PCR positive. Samples classified as *Brucella* spp. positive were then subjected to a multiplex speciation assay^22^ with oligonucleotide primers and probes detecting specific IS*711* insertions downstream of the *alkB* gene for *B. abortus* and downstream of the BMEI1162 locus for *B. melitensis*. Speciation assays were run using identical reaction volumes and conditions as described above on an ABI 7500 thermocycler. All samples, no template control, *B. abortus* and *B. melitensis* positive controls were tested in duplicates in all the runs. Any sample with amplification and a cycle threshold (Ct) value < 40 with the respective target was classified as positive for *B. abortus* or *B. melitensis*.

### Rose Bengal Test (RBT)

Animal and human serum samples were tested for antibodies against *Brucella* spp. using the RBT. The test was conducted, with the rapid slide agglutination suspension of *B. abortus* biovar 1 Weybridge strain No. 99 antigen (RSA-RB, IDVet, France). Briefly, the antigen and serum samples were brought to room temperature in a biosafety cabinet and vortexed. Thereafter, 25 µl of each serum sample (for cattle and camels) or 30 µl (for goat, sheep, and human sera) were mixed with an equal volume of antigen on the glossy side of a white tile using a clean, wooden splint. The tile was then gently rocked at room temperature for four minutes before observing agglutination under natural light. Any sample that had visible agglutination after four minutes was considered as RBT positive^23^.

### Data management and analysis

All the data were merged and cleaned in MS Excel version 2018 (IBM, California). Further analyses were performed using R statistical software version 3.6.3^24^. Descriptive analyses were done using the aggregated data from both sites. Analyses of associations between *Brucella* spp. PCR test status in animal and human populations were performed using mixed effects logistic regression models created with the function *glmer* in the package *lme4*^25^ with sampling location included as a random effect, and the data being specified as having binomial distribution. Variables evaluated in the human model to assess association with PCR status included age category, gender and RBT results. For livestock, variable history of abortion, RBT status, animal spp. and history of retained placenta were included in the model as potential predictors for *Brucella* PCR status. In this model male ruminants and camels were excluded due to absence of appropriate clinical history (abortion and retained placenta) and low numbers of observations of the assessed variables, respectively.

For analysis of *B. abortus* and *B. melitensis* outcomes data from humans and livestock were combined to evaluate the influence of host species on *Brucella* species detection. The percentage positive for each test by species and site was calculated and plotted, with binomial exact confidence intervals. Both models were fitted with sampling location included as a random effect, and the data being specified as having binomial distribution.

The maximal models were simplified using a likelihood ratio tests, with a p-value of ≤ 0.05 being considered statistically significant to get the final models. The intra-cluster correlation coefficients (*icc*) for within-location clustering of brucellosis for both livestock and humans were calculated from the variance components of the final multivariable models using the *icc* function in *sjstats* package^26^.

### Ethics approval and consent to participate

The research approval for this study was granted by the National Commission for Science, Technology, and Innovation (Ref. no. NACOSTI/P/19/81438/29438). Additional approvals for both livestock and human components of the study were provided by ILRI’s Institutional Animal Care and Use Committee (Ref. no. ILRI-IACUC2018-16) and Research Ethics Committee (Ref. no. ILRI-IREC2018-14), respectively. ILRI is recognized by the National Commission for Science and Technology in Kenya (NACOSTI/NBC/AC/01813) and internationally by the Federation wide assurance (number FWA00026536) to review and approve research studies. The research was performed in accordance with the relevant guidelines and regulations prescribed by the above research regulatory committees. For human subjects, a written informed consent was sought and obtained from all the adult participants (18 years and above), while parent’s consent was obtained for participants below the age of 12 years and both the parent or guardian consent together with the subject’s assent obtained for those between 13–17 years of age. The livestock owners also provided informed consent before inclusion into the study.

## Results

### Livestock population summary, RBT and *Brucella* spp. PCR results

The demographic characteristics of the sampled livestock are summarized in Table 1. A total of 597 livestock including 228 cattle, 162 goats, 158 sheep were sampled from Narok and Marsabit Counties combined. Camels (n = 49) were sampled in Marsabit County only, as these animals were not kept in Narok County. Most samples were collected from female animals (92%) (Table 1). Both IS711 and bcsp31 targets detected *Brucella* spp. in our samples (supplementary table S3). The results of the RBT and the genus-specific *Brucella* spp. PCR are shown in Table 1. The overall RBT positivity in livestock was 23.3% in cattle; 8.3% in goats; 5.7% in sheep; and 9.3% in camels (Table 1). The overall PCR positivity of *Brucella* spp. detected by PCR in livestock was 18.9% in cattle; 38.3% in goats; 38% in sheep; and 69.4% in camels (Table 1). The proportion of animals with a history of retained placenta was 2% and those with previous abortion was 12% (Table 1).

**Table 1:** Summary of livestock population composition, descriptive characteristics, RBT results and *Brucella* spp. PCR results.

Variable	Category	Rose Bengal results			PCR results		
		Total	No RBT positive	% RBT positive (95% CI)	Total	Number PCR positive	% *Brucella* spp. PCR positive (95% CI)
Species	Cattle	228	55	24.1 (18.7–30.2)	228	43	18.9 (14.0–24.6)
	Goats	158	38	8.2 (4.5–13.7)	162	62	38.3 (30.8–46.2)
	Sheep	138	8	5.4 (2.3–10.4)	158	60	38.0 (30.4–46.0)
	Camels	41	4	9.8 (2.7–23.1)	49	34	69.4 (54.6–81.7)
Sex	Male	45	19	13.3 (5.1–26.8)	45	19	42.2 (27.7–57.8)
	Female	530	74	14.0 (11.1–17.2)	552	180	32.6 (28.7–36.7)
Abortion	No	508	63	12.4 (9.7–15.6)	527	164	31.1 (21.2–35.3)
	Yes	67	17	25.4 (15.5–37.5)	70	35	50.0 (37.8–62.2)
Retained placenta	No	568	78	16.0 (12.9–19.6)	585	192	33.3 (29.5–37.3)
	Yes	10	2	25.0 (3.2–65.1)	12	7	63.6 (30.8–89.0)
Total	Total samples	575	80	13.9 (11.1–17.0)	597	199	33.3 (29.6–37.3)

*PCR* polymerase chain reaction, *CI* confidence interval, *RBT* Rose Bengal Test.

### Human population summary, RBT and *Brucella* spp. PCR results

A total of 257 humans were sampled (110 in Marsabit and 147 in Narok). Majority of samples were collected from female participants (57.6%). The age of human participants ranged from 3 to 96 years with the mean age of 32.6 years. Participant age was converted into a categorical variable with 3 levels for analysis: ≤ 20 years, 21–40 and > 40 years.

The overall PCR positivity of *Brucella* spp. in humans was 40.1% (95% CI 32.5–44.8), while the RBT positivity was 19.3% (95% CI 14.5–24.9, n = 238) (Table 2).

**Table 2:** Summary of human population descriptive, RBT results and *Brucella* spp. PCR results among suspected brucellosis patients referred for testing at the sampled medical facilities.

Variable	Category	Narok		Marsabit		Combined human data		
		Total tested	Number PCR positive	Total tested	Number PCR positive	Total tested	Number PCR positive	% *Brucella* spp. PCR positive (95% CI)
Age category (years)	≤ 20	39	13	18	2	58	16	27.6 (16.7–40.9)
	21–40	65	36	54	24	99	52	52.5 (42.2–62.7)
	> 40	23	8	10	3	54	17	31.5 (19.5–45.6)
RBT result	Negative	116	31	76	25	192	58	30.2 (26.2–34.4)
	Positive	48	12	15	7	46	20	43.5 (28.9–58.9)
Gender	Male	55	22	54	18	109	40	36.7 (27.7–46.4)
	Female	92	38	56	21	148	59	39.9 (31.9–48.2)
Total (site)	Total samples	147	60	110	39	257	99	38.5 (32.5–44.8)

*PCR* polymerase chain reaction, *CI* confidence interval, *RBT* Rose Bengal Test.

### Factors associated with *Brucella* spp. PCR status in humans

The final model fitted for human *Brucella* spp. PCR status only had age category as a significant variable positively associated with *Brucella* PCR status, LRT χ^2^ = 9.8 (Table 3). Individuals aged 21–40 were more likely to be PCR positive than individuals with an age category of less or equal to 20 years (Table 3). Sex and RBT status were not significantly associated with PCR status, therefore, we dropped the variables from the final model. The *icc* for within-location clustering of human brucellosis estimated for this model was < 0.001 (0.0–0.9).

**Table 3:** Summary of the final mixed-effects logistic regression models run to assess associations between variables and *Brucella* spp. PCR status in human.

Variables	Category (years)	Odds ratio (95% CI)	Odds ratio *P*-value	LRT χ^2^	LRT *p*-value	Df
Age category	≤ 20	1 (baseline)		9.81	0.03	2
	21–40	2.8 (1.2–6.6)	0.016			
	> 40	1.2 (0.4–3.1)	0.771			

*CI* confidence interval, *LRT* χ^2^ Likelihood Ratio Test Chi-square value, P-value according to Pearson Chi-square test, *Df* degrees of freedom.

### Factors predicting *Brucella* spp. PCR positivity in livestock

There was a positive and significant relationship between RBT status and *Brucella* PCR status, with RBT seropositive animals having elevated odds (OR = 8.3, 95% CI 4.0–17.1) of testing positive by PCR. Goats and sheep had higher odds (OR = 2.6, 95% CI 1.3–5.2) and (OR = 2.2, 95% CI 1.1–4.6) respectively of being PCR positive for *Brucella* spp. compared to cattle (Table 4).

**Table 4:** Summary of multivariable mixed-effects logistic regression models run to predict *Brucella* spp. PCR status in livestock.

Variables	Category (years)	Odds ratio (95% CI)	Odds ratio *P*-value	LRT χ^2^	LRT *p*-value	Df
Animal species	Cattle	1 (baseline)		7.99	0.018	2
	Goats	2.6 (1.3–5.2)	0.005			
	Sheep	2.2 (1.1–4.6)	0.036			
RBT	Positive	8.3 (4.0–17.1)	< 0.001	35.98	< 0.001	1

Location *icc* = 0.4.

*CI* confidence interval, P-value according to Pearson Chi-square test, *Df* degrees of freedom, *RBT* Rose Bengal Test, *icc* intra-cluster correlation coefficients.

### Detection of *Brucella abortus* and *Brucella melitensis*

Of 298 *Brucella* spp. PCR positive livestock and human samples, 117 (39.3%) were positive for the *B. abortus* specific target, 111 (37.2%) were positive for the *B. melitensis* target and 68 (22.8%) did not amplify with either *B. abortus* or *B. melitensis* primer targets. No samples amplified with more than one target. The distribution of *Brucella* spp. differed in the two study sites, with *B. abortus* detected in most typed samples from Marsabit 50 (67.7.0%, 95% CI 54.8–77.1), whereas *B. melitensis* was detected in majority of typed samples from Narok 54 (65.9%, 95% CI 54.6–76.0).

*Brucella abortus* was detected in all species with the highest positivity in camels (64.7%), followed by cattle (60.5%), humans (44.4%), goats (24.2%), and sheep (20.0%). Sheep and goats had a significant reduced odds of association OR = 0.2, 95% CI 0.1–0.5 and OR = 0.2, 95% CI 0.0.6, with *B. abortus* compared to cattle, while the lower odds observed in camels and humans were not significant when compared with cattle (Table 5).

**Table 5:** Summary of mixed-effects logistic regression models of *Brucella abortus* status in different host species.

Level	Positivity for targeted *Brucella* spp.		Univariable mixed-effects logistic regression				
Host	n/N	% positivity and (95% CI)	Odds Ratio 95% CI	Odds ratio *P*-value	LRT χ^2^	LRT p-value	LRT df
Cattle	26/43	60.5 (44.4–75.0)	Baseline		16.71	0.002	4
Goats	15/62	24.2 (14.2–36.7)	0.2 (0.1–0.6)	0.003			
Sheep	12/60	20.0 (10.8–32.3)	0.2 (0.1–0.5)	0.001			
Camel	22/34	64.7 (46.4–80.3)	0.9 (0.3–2.7)	0.916			
Human	44/99	44.4 (34.5–54.8)	0.5 (0.2–1.0)	0.055			

Location *icc* = 0.10.

*CI* confidence interval, LRT χ^2^ Likelihood Ratio Test Chi-square value, P-value according to Pearson Chi-square test, *n* number of positives, *N* total number tested.

*Brucella melitensis* was found in the highest proportion in sheep (63.3%), followed by goats (50.0%), humans (29.3%), camel (22.2%), and cattle (16.3%). The mixed-effects model found that sheep had higher odds of being associated with *B. melitensis* OR = 1.8, 95% CI 0.8–3.8*,* while cattle, camels and humans had lower odds OR = 0.2, 95% CI 0.1–0.6, OR = 0.3, 95% CI 0.1–0.9, and OR = 0.5, 95% CI 0.3–1.2 compared to goats respectively (Table 6).

**Table 6:** Summary of mixed-effects logistic regression models of *Brucella melitensis* status in different host species.

Level	Positivity for targeted *Brucella* spp.		Univariable mixed-effects logistic regression				
Host	n/N	% positivity and (95% CI)	Odds Ratio 95% CI	Odds ratio *P*-value	LRT χ^2^	LRT p-value	LRT df
Goats	31/62	50.0 (37.0–63.0)	Baseline		22.98	< 0.001	4
Cattle	7/43	16.3 (06.8–30.7)	0.2 (0.1–0.6)	0.003			
Sheep	38/60	63.3 (49.9–75.4)	1.8 (0.8–3.8)	0.134			
Camel	6/34	17.6 (06.8–34.5)	0.3 (0.1–0.9)	0.037			
Human	29/99	29.3 (20.6–39.3)	0.5 (0.3–1.2)	0.177			

Location *icc* = 0.05.

*CI* confidence interval, LRT χ^2^ Likelihood Ratio Test Chi-square value, P-value according to Pearson Chi-square test, *n* number of positives, *N* total number tested.

**Figure 2:**
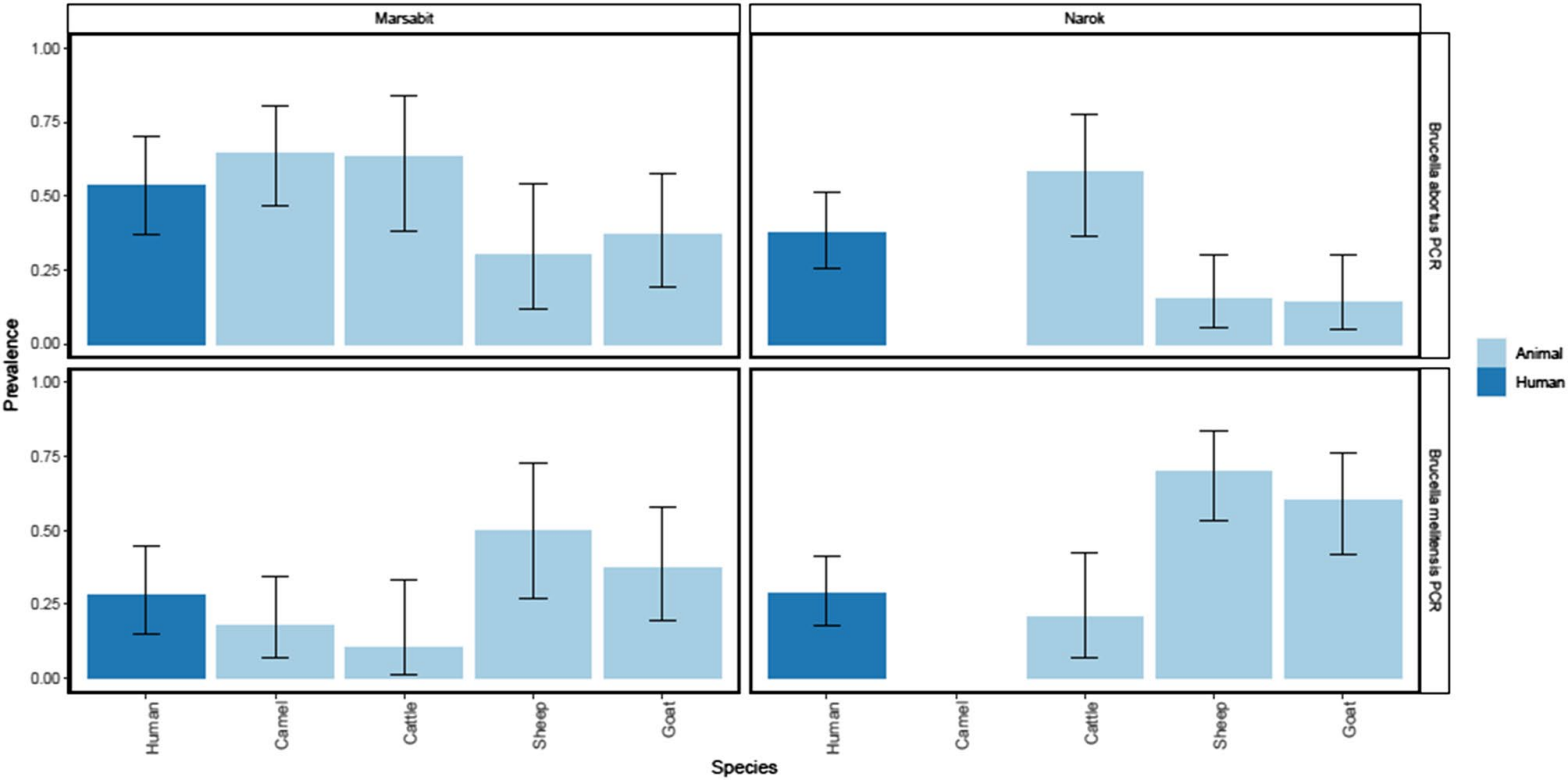
Graphical presentation of the proportion of each host species testing positive for *B. abortus* and *B. melitensis* in Narok and Marsabit. The four panels show data for each site and *Brucella* species respectively. In each panel the bars indicate the proportion of each species testing positive. The error bars indicate exact binomial confidence intervals.

Analysis of *Brucella* spp. detected in different animal hosts in Marsabit and Narok gave a similar distribution trend that is comparable to those observed in the combined data (Fig. 2).

## Discussion

This molecular study on the epidemiology of *Brucella* spp. in various host species has highlighted some key findings: we detected *Brucella* DNA in serum from humans and all the livestock species studied. This study also identified RBT positive status as a significant predictor for *Brucella* PCR positivity in livestock, while age category was associated with *Brucella* PCR positive status in humans. Finally, both *B. abortus* and *B. melitensis* were detected in humans and all the livestock species included in the study.

The detection of *Brucella* in four different animal hosts indicates a complex epidemiology. In this study, we have demonstrated that camels have a higher proportion of *Brucella* PCR positives than other species. This could partly be attributed to the high frequencies of migration of camel herds^27^ that enhance sharing of grazing areas and watering points or direct interactions between herds, thereby increasing their exposure to infectious pathogens such as *Brucella.* Detection of zoonotic species *Brucella* (*B. abortus* and *B. melitensis*) in camels could present a public health problem, given the steady rise in camel populations in Kenya^28^, primarily for milk production^29–31^. Consumption of raw milk^9^, and the significant increase in production of camel milk^29, 32^ and the extensive reach of camel milk value chains further highlight the need for more investigations and intervention programs to control the potential role of camels in transmission of brucellosis to humans, and other possible zoonotic diseases.

People within the age of 21–40 years were more likely to get positive *Brucella* PCR status. Earlier studies have also reported higher prevalence rate in humans within the similar age category^33, 34^. Based on observation made during field data collection, the high positivity in the age group 21–40 years could be attributed to their primary responsibility of herding, milking, and helping animals during parturition. Thus, having the highest exposure compared to the younger population (below 21 years) that are either school going or have less contact when taking care of animals. Those above 40 years of age tend to take more leadership roles as they reduce their active involvement in taking care of animals, hence reducing their risk of acquiring zoonotic infection through direct contact with the animals or contaminated animal products. This finding is comparable to earlier studies^35^ that reported high prevalence of brucellosis in the same age category and associated this to their occupational roles with livestock. The distribution of human cases across all age groups points to consumption of contaminated animal products as an alternative transmission route as observed by other studies within the region^36, 37^.

Our study found out that the odds of *Brucella* PCR positive status were eight times higher in animals with RBT positive results compared with those with negative results. This finding agrees with earlier recommendation that RBT is an appropriate test to be used for diagnosis of brucellosis and may be a useful screening test in low resource regions where PCR testing is constrained^38^. However, these two tests detect different markers of infection and even though complementary, they give overlapping but different information^39^. The low sensitivity of RBT observed in this study, and previous studies^40–42^ makes it necessary for it to be considered alongside other serological tests such as ELISA, slow Agglutination Tests (SAT) the Fluorescence Polarization Assay (FPA) and Complement Fixation Tests (CFT)^43–45^. This study however, exploited quantitative real-time PCR assays to detect *Brucella* DNA extracted from serum samples of the various hosts. Real-time PCR assays have been recommended as rapid and sensitive tests for detection of *Brucella* spp.^21, 46^. In this study, we tested DNA extracted from serum samples for *Brucella* positivity. Serum has been previously shown to be a good sample type for *Brucella* DNA detection in livestock and humans^47^. The exploitation of the same sample type for both serological testing (to advise *Brucella* exposure), and direct nucleic acid detection is a growing field of brucellosis diagnosis^47–49^. The added convenience of bypassing the need for tedious and dangerous culture procedures makes PCR a powerful brucellosis surveillance tool. Future studies should focus on generating more data to explore the agreement between serology and molecular approaches, and how each of the approaches informs about infection status in animals and humans.

Location was used as a random effect in our analysis to account for the potential effect of clustering of *Brucella* PCR positive cases in the models. The *icc* for location was estimated to be 0.4 for the livestock dataset. This is an indication that brucellosis infection in livestock tends to cluster within locations, which may be due to close or frequent interaction of herds within the same location. However, the human PCR positive cases were not clustered by locations (*icc* = 0). This may be attributed to the fact that humans may not only get exposed to *Brucella* infection within their residential location but could also acquire infection in other locations through consumption of contaminated animal products and contact with infected animals or their products. The broader distribution of milk and other dairy products in Kenya^50^ may also contribute to the lack of clustering of brucellosis cases in humans.

Both *B. abortus* and *B. melitensis* were detected in all livestock species. Our findings of both species of the pathogen across all hosts is consistent with earlier reports that *Brucella* spp. are not host specific and that cross transmission of *Brucella* spp. from one livestock host to the other could be occurring in areas with close interaction between different animal species^14^. Congregation of animals around communal watering points, keeping of mixed herds, and sharing of grazing sites have been reported to increase chances of brucellosis transmission^10^. Therefore, this could have contributed to the cross transmission of *Brucella* spp. observed in the two pastoral areas studied. We identified *B. abortus* most commonly in cattle and camels, while infection in sheep and goats were mainly associated with *B. melitensis*. This finding is consistent with existing knowledge on the preferential nature of *Brucella* spp. in the different animal hosts^51^.

Brucellosis infection in sheep and goats with *B. melitensis* is endemic in East Africa with earlier studies in Kenya in 1972^52^ and 1976^53^, in Tanzania 1967^54^, and 1996^55^ reporting presence of *B. melitensis* in the region. Earlier studies in East Africa also reported circulation of *B. abortus* in cattle in Kenya 2002^56^, in Tanzania^57, 58^ and in Uganda^59^. This study is among the first reports of *Brucella* spp. circulating in camels in Kenya, although infection of camels with *B. abortus* has also been reported in Sudan^60^ and Pakistan^61^.

Our study found that *B. abortus* and *B. melitensis* are detectable in humans with clinical suspicion of brucellosis in Narok and Marsabit. Previous findings in the wider region, also reported the presence of *B. abortus* and *B. melitensis* in the human population^62, 63^. Human infection with *Brucella* spp. may be transmitted from multiple livestock species, given that *B. abortus* and *B. melitensis* detected in humans were also found in cattle, sheep, goats, and camels kept in the same locality. Brucellosis control programs aimed at reducing human brucellosis should therefore target all livestock hosts studied.

Abortions and retained placenta were rarely reported in our study, therefore our ability to assess their association with *Brucella* PCR status was limited by the low numbers of observations. The targeted study design limits the power of inference to population level prevalence but could have also contributed to the high PCR positivity reported in the study. The PCR speciation assay used only targeted *B. abortus* and *melitensis*. Therefore, 22.8% of the genus *Brucella* PCR positive samples that did not amplify with our two targets could not be identified, and other *Brucella* species circulating in the targeted population might have been missed. Future studies could focus on the known risk factors for positivity, more epidemiologically focused study designs and improved typing options using real-time PCR to address some of these prevailing challenges in the molecular epidemiology of brucellosis in the region.

## Conclusion

This is among the first studies in the region to undertake a population level molecular study aimed at detecting circulating species of *Brucella* in several livestock hosts and humans. This study provided evidence of the presence of *B. abortus* and *B. melitensis* in multiple livestock species and humans in Kenya. Future studies should consider expanding the range of real-time PCR typing options to shed more light on all the *Brucella* species present in the targeted population. Our findings also confirmed that *B. melitensis* have a significant positive association with sheep and goats, while *B. abortus* is associated with cattle and camels. Cross transmission of different *Brucella* spp. between different animal hosts and the potential of human infection being caused by multiple livestock hosts was also highlighted. Brucellosis control programs in Kenya should use a One Health strategy targeting multiple host species.

## Supplementary Information

Supplementary Information 1.

Supplementary Information 2.

Supplementary Information 3.

Supplementary Information 4.

## Data Availability

All the data are included in this article and its supplementary files.
