# Supplementary material for: Molecular epidemiology of Brucella species in mixed livestock-human ecosystems in Kenya
Source: Sci Rep. 2021 Apr 23;11:8881. doi: 10.1038/s41598-021-88327-z (PMC8065124; doi:10.1038/s41598-021-88327-z)
Supplement: Supplementary file 1 — Supplementary Information 1. [file 41598_2021_88327_MOESM1_ESM.docx]

**Molecular epidemiology of *Brucella* species in mixed livestock-human ecosystems in Kenya**

James M. Akoko*^1,2,3^, Roger Pelle^2^, AbdulHamid S. Lukambagire^4^, Eunice M. Machuka^2^, Daniel Nthiwa^5^, Coletha Mathew^4^, Eric M. Fèvre^3,6^, Bernard Bett^3^, Elizabeth A. J. Cook^3,6^, Doreen Othero^7^, Bassirou Bonfoh^8^, Rudovick Kazwala^4^, Gabriel Shirima^9^, Esther Schelling^10^, Jo E.B. Halliday^11^, Collins Ouma^1^

^1^Department of Biomedical Sciences and Technology, Maseno University, Kenya; **^2^Biosciences eastern and central Africa - International Livestock Research Institute (BecA-ILRI) Hub KE**, Kenya; ^3^International Livestock Research Institute; Kenya; ^4^Sokoine University of Agriculture, Tanzania; ^5^Department of Biological Sciences, University of Embu, Embu, Kenya; ^6^Institute of Infection, Veterinary and Ecological Sciences, University of Liverpool, UK; ^7^Department of Public Health, Maseno University, Kenya; ^8^Centre Suisse de Recherches Scientifiques en Côte d’Ivoire; ^9^Nelson Mandela African Institute of Science and Technology, Tanzania; ^10^**Vétérinaires sans Frontières Suisse CH,** Switzerland; ^11^Institute of Biodiversity, Animal Health and Comparative Medicine, College of Medical Veterinary and Life Sciences, University of Glasgow, Glasgow G12 8QQ, United Kingdom.

* Corresponding author (email address; [jamesakoko@yahoo.com](mailto:jamesakoko@yahoo.com))

**S1. Standard curve with *Brucella* positive controls, showing the efficiency of our assay**

1. **Positive controls received**

1 µg vacuum dried purified DNA of:

| **Brucella species** | **Biotype** | **Strain** |
| --- | --- | --- |
| Brucella abortus | Biotype 1 | Reference strain 544 |
| Brucella melitensis | Biotype 1 | Reference strain 16M |

1. **Reconstitution and Dilution**

Reconstitution of the controls stock DNA involved addition of 100 µl of RNase-DNase free water to the lyophilized samples. The final concentration of stock DNA was 10ng/µl.

10-fold serial dilutions were prepared for the working concentration and for generation of a standard curve at qPCR.

1. **qPCR Results**

The multiplex reaction worked as expected. No contamination was observed in the negative controls. All the targets (genus *Brucella*, *B. melitensis*, and *B. abortus*) had amplifications as below;

**Standard curve for the genus *Brucella* target (Bcsp31)**


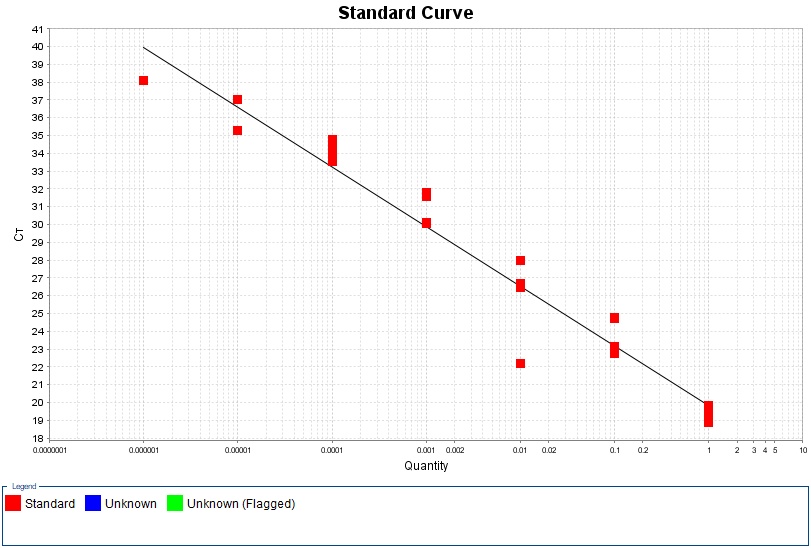


**Figure S2_figure 1.** The genus *Brucella* target standard curve. Efficiency (Eff%: 98.748, R^2^: 0.956, y = -3.35x + 19.839

**Standard curve for *B. abortus* controls**


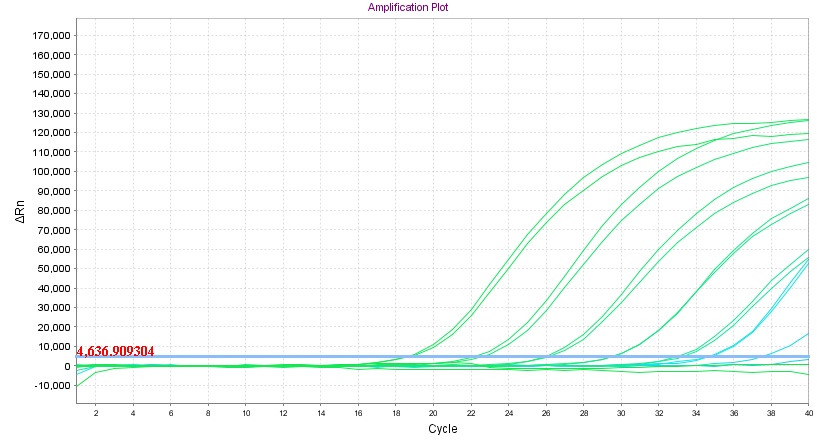


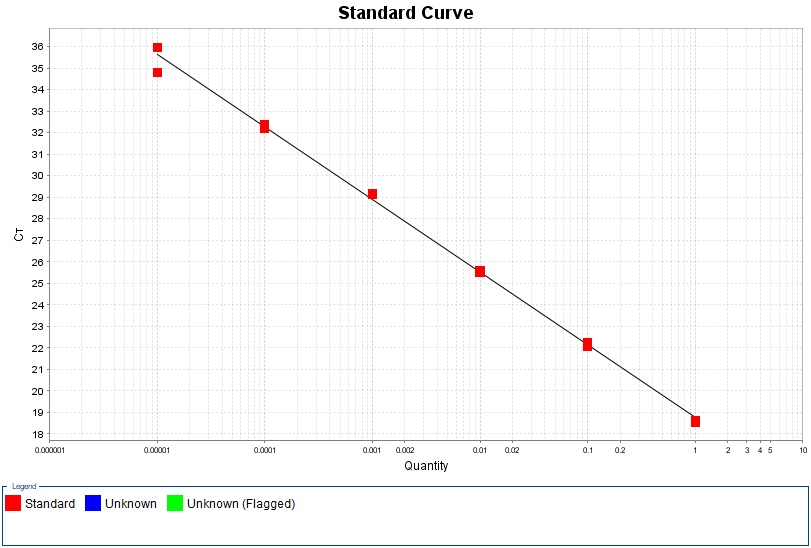


**S2_figure 2**: *B. abortus* amplification curve and standard curve. qPCR efficiency (Eff%: 98.004, R^2^: 0.997, y=-3.371x +18.759)

**Standard curve for *B. Melitensis***


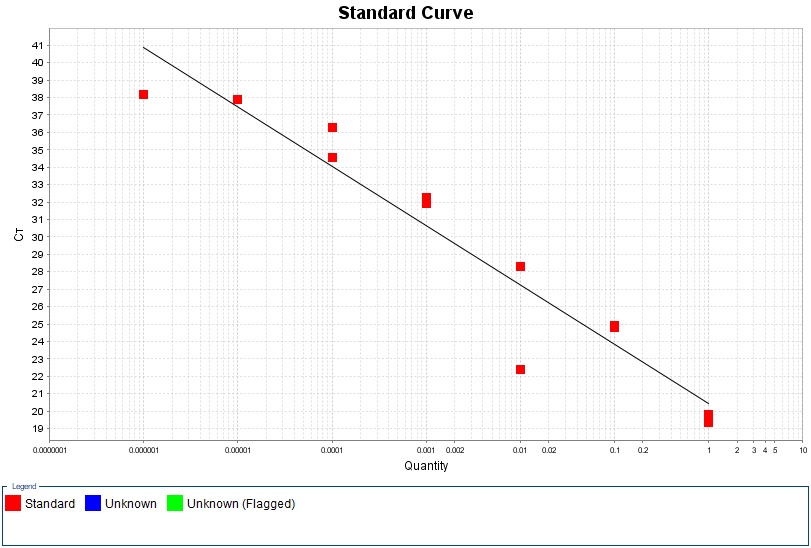


**S2_figure 3**: B. melitensis standard curve. qPCR efficiency (Eff%: 96.448, R^2^: 0.914, y=-3.41x +20.413)

1. **Conclusion**

The diagnostic assay for the detecting the genus *Brucella*, *B. abortus* and *B. melitensis* were optimal.
