# Supplementary material for: Molecular epidemiology of Brucella species in mixed livestock-human ecosystems in Kenya
Source: Sci Rep. 2021 Apr 23;11:8881. doi: 10.1038/s41598-021-88327-z (PMC8065124; doi:10.1038/s41598-021-88327-z)
Supplement: Supplementary file 2 — Supplementary Information 2. [file 41598_2021_88327_MOESM2_ESM.docx]

**Molecular epidemiology of *Brucella* species in mixed livestock-human ecosystems in Kenya**

James M. Akoko*^1,2,3^, Roger Pelle^2^, AbdulHamid S. Lukambagire^4^, Eunice M. Machuka^2^, Daniel Nthiwa^5^, Coletha Mathew^4^, Eric M. Fèvre^3,6^, Bernard Bett^3^, Elizabeth A. J. Cook^3,6^, Doreen Othero^7^, Bassirou Bonfoh^8^, Rudovick Kazwala^4^, Gabriel Shirima^9^, Esther Schelling^10^, Jo E.B. Halliday^11^, Collins Ouma^1^

**S2. Oligonucleotide primers and probes used to perform PCR assays**

| Target | Targeted gene | Sequences of primers and probes (5’ -3’) | Fluorophore/ quencher | Reference |
| --- | --- | --- | --- | --- |
| Genus *Brucella* | Bcsp31 | Probe: AAATCTTCCACCTTGCCCTTGCCATCA Forward: GCTCGGTTGCCAATATCAATGC  Reverse: GGGTAAAGCGTCGCCAGAAG | 6-FAM/BHQ1 | Probert 2004 |
| Genus *Brucella* | IS711 | Probe: AAG CCA ACA CCC GGC  Forward: GGC CTA CCG CTG CGA AT  Reverse: TTG CGG ACA GTC ACC ATA ATG | FAM/-MGBNFQ | *Matero,*  *2011* |
| *B. melitensis* | IS711 downstream of BMEI1162 | Probe CAGGAGTGTTTCGGCTCAGAATAATCCACA Forward AACAAGCGGCACCCCTAAAA  Reverse CATGCGCTATGATCTGGTTACG | Texas Red/BHQ2 | Probert 2004 |
| *B.* *abortus* | IS711 downstream of alkB | Probe: CGCTCATGCTCGCCAGACTTCAATG Forward: GCGGCTTTTCTATCACGGTATTC  Reverse: CATGCGCTATGATCTGGTTACG | JOE/BHQ1 |  |
