# Supplementary material for: Molecular epidemiology of Brucella species in mixed livestock-human ecosystems in Kenya
Source: Sci Rep. 2021 Apr 23;11:8881. doi: 10.1038/s41598-021-88327-z (PMC8065124; doi:10.1038/s41598-021-88327-z)
Supplement: Supplementary file 3 — Supplementary Information 3. [file 41598_2021_88327_MOESM3_ESM.docx]

**Molecular epidemiology of *Brucella* species in mixed livestock-human ecosystems in Kenya**

James M. Akoko*^1,2,3^, Roger Pelle^2^, AbdulHamid S. Lukambagire^4^, Eunice M. Machuka^2^, Daniel Nthiwa^5^, Coletha Mathew^4^, Eric M. Fèvre^3,6^, Bernard Bett^3^, Elizabeth A. J. Cook^3,6^, Doreen Othero^7^, Bassirou Bonfoh^8^, Rudovick Kazwala^4^, Gabriel Shirima^9^, Esther Schelling^10^, Jo E.B. Halliday^11^, Collins Ouma^1^

**S3. Showing the number of samples detected by each *Brucella* genus target (Bcsp31 and IS711) and those that amplified with both targets.**

| Target | positive | Negative | Total |
| --- | --- | --- | --- |
| IS711 | 351 | 503 | 854 |
| Bcsp31 | 299 | 555 | 854 |
| Both target | 298 | 556 | 854 |
