# Supplementary material for: Molecular epidemiology of Brucella species in mixed livestock-human ecosystems in Kenya
Source: Sci Rep. 2021 Apr 23;11:8881. doi: 10.1038/s41598-021-88327-z (PMC8065124; doi:10.1038/s41598-021-88327-z)

**Molecular epidemiology of *Brucella* species in mixed livestock-human ecosystems in Kenya**

James M. Akoko*^1,2,3^, Roger Pelle^2^, AbdulHamid S. Lukambagire^4^, Eunice M. Machuka^2^, Daniel Nthiwa^5^, Coletha Mathew^4^, Eric M. Fèvre^3,6^, Bernard Bett^3^, Elizabeth A. J. Cook^3,6^, Doreen Othero^7^, Bassirou Bonfoh^8^, Rudovick Kazwala^4^, Gabriel Shirima^9^, Esther Schelling^10^, Jo E.B. Halliday^11^, Collins Ouma^1^

S4. Showing performance of RBT Vs PCR on samples collected from different animal species in Narok and Marsabit


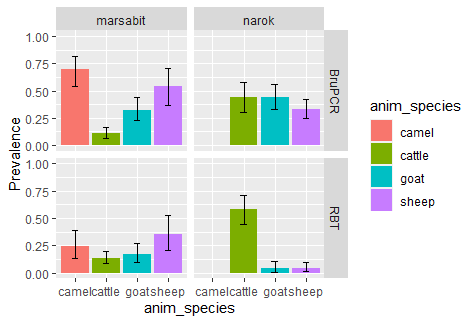

Supplement: Supplementary file 4 — Supplementary Information 4. [file 41598_2021_88327_MOESM4_ESM.docx]
